# Prospective comparative study of the effects of lidocaine on urodynamic and sensory parameters in bladder pain syndrome

**DOI:** 10.1007/s00192-019-03892-2

**Published:** 2019-03-14

**Authors:** Ifeoma Offiah, Elaine Dilloughery, Stephen B. McMahon, Barry A. O’Reilly

**Affiliations:** 1 Department of Urogynaecology, Cork University Maternity Hospital, Wilton, Co. Cork, Ireland; 2 Neurorestoration Group, Wolfson CARD, King’s College London, Hodgkin Building, Guy’s Campus, London, SE1 1UL UK; 3 University Hospitals Plymouth, Derriford Hospital, Derriford Road, Plymouth, PL6 8DH UK; 4 Department of Urogynaecology, ASSERT Centre, University College Cork, Cork, Ireland; 5 Department of Obstetrics and Gynaecology, University College Dublin, Dublin, Ireland

**Keywords:** Bladder pain syndrome, Central sensitisation, Peripheral pain modulation, Lidocaine, Urodynamics

## Abstract

**Introduction and hypothesis:**

Intravesically administered lidocaine is used in patients with bladder pain syndrome (BPS) to test the hypothesis that symptoms have a peripheral versus central mechanism.

**Methods:**

A cross-sectional study of 24 female patients with BPS was performed. The Central Sensitisation Inventory (CSI) and Kings Health Questionnaire (KHQ) were completed. Urodynamic assessment was undertaken. Women were asked to report their pain using a numeric rating scale at cystometric capacity and post void. Participants then received an intravesical instillation of either 20 ml of 2% alkalinised lidocaine (*n* = 16) or 20 ml of normal saline (*n* = 8). These solutions were allowed to remain in situ for 20 min and pain score repeated. Urodynamics was repeated.

**Results:**

There was a statistically significant volume increase following lidocaine treatment: maximal cystometric capacity (MCC) 192–261 ml post lidocaine (*p* = 0.005.) In contrast, there was no significant difference in the saline controls: MCC 190–183 ml (*p* = 0.879.) Individual analysis revealed five of 16 lidocaine participants did not respond to lidocaine. These five reported a significantly worse quality of life (QoL) than lidocaine responders and had a tendency towards central sensitivity syndromes.

**Conclusion:**

Lidocaine significantly improved MCC in 11/16 participants in this study. These patients appear to have peripherally mediated disease. However, the failure of response to treatment in five participants, as well as their tendency towards central sensitivity syndromes, implies that in this subgroup, a peripheral drive from the bladder is not critical to their pain, suggesting central nervous system (CNS) pathology. This simple and safe test could be used to stratify patients for research or therapeutic trials.

**Electronic supplementary material:**

The online version of this article (10.1007/s00192-019-03892-2) contains supplementary material, which is available to authorized users.

## Introduction

Bladder pain syndrome (BPS), also known as interstitial cystitis (IC), is a heterogeneous chronic pelvic pain condition. It is defined by the International Continence Society (ICS) as “the complaint of suprapubic pain related to bladder filling, accompanied by other symptoms such as frequency or nocturia in the absence of other proven urinary infections or other obvious pathology” [1]. This diagnosis is heavily dependent on patient history of bladder pain in the absence of confounds arising from other disorders affecting the lower urinary tract. Diagnosis is therefore often delayed, whilst alternatives are excluded [2]. Once diagnosed, many patients often try multiple treatment options before finding one that gives them significant or any relief [3]. However, despite excellent care, some patients never find effective relief of their pain, even following extreme interventions such as cystectomy or urinary diversion, when phantom bladder pain or sensations of bladder fullness can persist [4, 5]. Risk factors for phantom pain include chronic pain, traumatic amputation and psychological factors.

There are multiple aetiologies about the mechanisms of BPS symptoms. However, one question is whether they are peripherally mediated or arise from abnormal processing of sensory information within the central nervous system (CNS) and become independent of peripheral inputs. There are precedents for both of these mechanisms in chronic pain states, but no consensus with BPS. The peripheral mechanism is abnormal activity or responsiveness in sensory neurones, and there is a large evidence base for a variety of mediators, often inflammatory mediators, producing this kind of change in peripheral nociceptors [6]. The central mechanisms may have a number of components. One is so-called central sensitisation, a potentiation of synaptic transmission of pain-related signals, best described in the spinal cord but also likely to occur elsewhere in the CNS [7]. Other CNS mechanisms are a change in the descending modulatory influences on the spinal processing of pain or the reorganisation of cortical networks that ultimately lead to pain sensation [8, 9]. Both of these can readily help explain abnormal sensory events, such as on-going pain and hyperalgesia, but in different patients, different mechanisms may predominate.

It is both of intellectual and practical interest to understand what is affecting patients with BPS. The practical benefit is that it would potentially help in selecting therapies for individual patients. In this study, the hypothesis that the pain of BPS is driven by a combination of both peripheral and central mechanisms is examined. Our aim was to evaluate the effect of intravesically administered local anaesthetics on perceived pain and urodynamic volumes in patients with BPS. Urodynamics, an objective measure of bladder functionality, is used to assess bladder response to lidocaine instillation in BPS. This is the gold standard examination for the assessment of lower urinary tract disorders. Urodynamics is used in this study to reduce participant performance bias and the placebo effect related to lidocaine treatment. In patients in whom the pain is centrally driven and independent of peripheral pathology, it is hypothesised that instillation of local anaesthetics will not have an effect on perceived pain or urodynamic volumes and bladder capacity.

## Materials and methods

This study was approved by the Cork Research Ethics Committee of the University College Cork and performed at the Cork University Maternity Hospital (CUMH) tertiary referral centre, a European Urogynaecological Association subspecialty-accredited centre in conjunction with the University of Cork. Analysis was performed at King’s College London. Written informed consent was obtained from all participants. Patients with a history of bladder pain accompanied by at least one other symptom, such as urinary frequency, urgency or nocturia, present for at least 3 months, as per the International Continence Society (ICS) definition of BPS, were recruited into the study [1]. Women with lidocaine hypersensitivity or other proven forms of cystitis such as infective, chemical or radiation cystitis, or neurological disease such as multiple sclerosis, Parkinson’s disease, spinal cord injury or neural tube defects, affecting bladder function were excluded. Also excluded were women with severely debilitating or concurrent medical or psychiatric conditions that could influence reporting. Women who were unable to void spontaneously, those receiving local anaesthetic analogue therapy or women with a history of chronic pelvic pain conditions such as endometriosis, were also excluded.

Participants completed the validated King’s Health Questionnaire (KHQ) that evaluates the impact of disease on patient quality of life (QoL) (Appendix 1). In addition, participants completed the Central Sensitisation Inventory (CSI), Part B Questionnaire, a validated patient-reported screening instrument formulated by the University of Texas, USA, to identify patients whose symptoms may be related to a central sensitivity syndromes (CSS) [10]. In the absence of any other scoring system in place for central sensitisation, this psychometric tool qualifies as a valuable screening instrument for clinicians to aid the identification of patients with CSS (Appendix 2).

Urodynamic assessment was performed to rule out detrusor overactivity (DO). Urodynamics evaluates the effect of bladder filling on bladder function and sensory processing. Since the pain of BPS manifests primarily during bladder filling, performing this study during urodynamic examination is the optimal approach for analysing the mechanisms of pain associated with BPS. Urodynamics was performed by the urogynaecology specialist midwife using the standardised ICS recommendations [11].

Two catheters were inserted: one into the bladder via the urethra and the second into the vagina. The bladder was filled at a rate of 80 ml/min using 0.9% normal saline, as per ICS recommendations [11]. During the filling phase, the following indices were recorded: first sensation, normal desire, strong desire, maximal cystometric capacity (MCC), and cystometric capacity (CC). MCC is defined as the volume at which the patient feels she can no longer delay micturition; CC is the bladder volume at the end of the filling; it is the volume voided plus any residual urine [11]. Subsequently, an 11-point numeric rating scale (NRS), which measures pain intensity from 0 (no pain) to 10 (worst possible pain), was performed at MCC and CC (Appendix 3). Bladders were emptied and the pain score repeated. Participants with any of the following urodynamic findings were allowed to continue with the investigation: low compliance as seen on urodynamic tracing, early sensations or bladder pain on filling (pain score > 5 on NRS at CC). Participants with no abnormality detected on urodynamics were excluded from the study at this stage, as they were deemed unlikely to have BPS with normal filling volumes and insignificant pain score at CC.

Subsequently, each study participant was randomly assigned to receive either 20 ml of 2% alkalinised lidocaine or 20 ml of 0.9% normal saline into their bladders. The lidocaine was alkalinised by the CUMH pharmacy department as follows: 3 ml of sodium bicarbonate dissolved in 9 ml of 0.9% saline mixed with 8 ml of 2% lidocaine. To assign either alkalinised lidocaine or saline, a pre-prepared note with alkalinised lidocaine or saline was picked from an envelope by the investigator. Participants were blinded to the treatment received. Solutions were instilled into the bladder using the already present urodynamic bladder catheter and allowed to remain in situ for 20 min, at the end of which the numeric pain score was again recorded. Finally, the urodynamic assessment as above was repeated. These urodynamic indices are applicable, consistent and reproducible and are thus used to evaluate response to instillation with either lidocaine or saline.

In this study, there was a discrepancy in the number of patients treated with lidocaine vs saline, with a 2:1 difference. This is because the main question evaluated was the difference in response to lidocaine bladder instillation. Urodynamic parameters are prone to accommodation. Hence, participants who received saline were present to demonstrate what no treatment with local anaesthetic should appear like on subsequent urodynamic distension: they therefore represent the control group. Rather than healthy controls, patients with BPS were chosen so comparisons could be made on the effects of saline and lidocaine on urodynamic distention patterns. Consequently, by studying the one disease state, variability in urodynamic test results could be reduced and saline controls used as a reference group.

Post urodynamics, all participants were referred for cystoscopy. Though hydrodistension is a therapeutic strategy in BPS management, in this study, cystoscopy was performed for diagnostic purposes, to check for BPS and to exclude any other bladder or urethral pathologies. While under general anaesthesia, the patient’s bladder was filled by placing a bag of saline 80 cm above the bladder and allowing distension under gravity. The European Society for the Study of IC/BPS (ESSIC) criteria for cystoscopic BPS diagnosis and assessment was followed, providing a structured scoring system for cystoscopic findings [12]. These include reduced bladder capacity, glomerulations, Hunner’s lesions and petechial haemorrhages.

Power analysis was conducted using a priori sample size calculator for Student’s *t* test. To reach a power of 80%, a sample size of 16 patients in the lidocaine group was calculated, with an alpha level of 0.05 and an effect size of 0.9 derived from previously reported data of lidocaine effect on BPS/IC by Nickel et al. [13]. Analysis using Student’s *t* test was performed for urodynamic tests volumes. Two-way analysis of variance (ANOVA) was used to compare patient groups and volumes. *P* value of <0.05 was deemed statistically significant. As pain scores are categorical data, the non-parametric Mann–Whitney *U* test was used to calculate pain scores on the numeric rating scale. Means with standard deviation (SD) are used to describe pain scores, and a frequency distribution of responses table is presented.

## Results

Forty-six female patients were screened for inclusion in the study following referral from the Gynaecology Outpatients Department. Thirty-four participants (74%) met inclusion and exclusion criteria. Twelve of the 46 patients (26%) recruited were not ultimately included either because they presented no features suggestive of BPS on urodynamics and excluded following the first urodynamic examination (*n* = 4) or had no cystoscopic evidence of disease (*n* = 8). A further ten (21.7% of total number screened for inclusion) were excluded following the first urodynamic examination, as they were unable to tolerate the pain associated with urodynamic distension and requested that they not proceed further with the protocol and be removed from the study. The remaining 24 participants had a history of bladder pain for at least 3 months. There was obvious correlation between patient age, parity, body mass index (BMI) and risk-factor assessment. No participant had concurrent lower urinary tract disorders or any other chronic pain disorders, and all midstream urine specimens were normal. All showed one or more signs suggestive of BPS on urodynamics and were allowed to complete the study; 16 were assigned to receive alkalinised lidocaine and eight normal saline. All participants had evidence of BPS on cystoscopy.

### Pain score

**Fig. 1:**
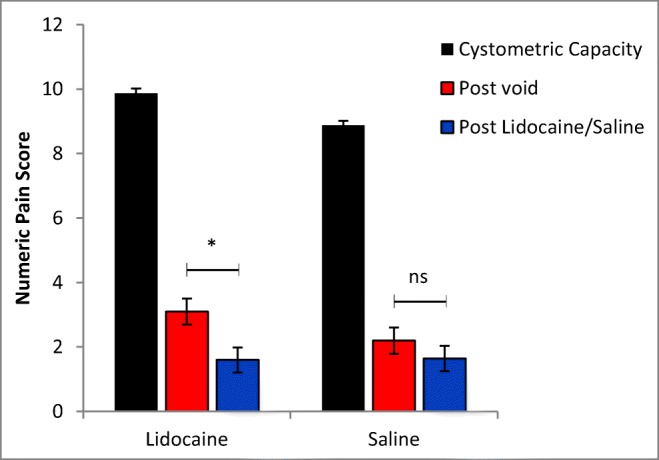
Mean numeric pain score at cystometric capacity, post void and post lidocaine or saline treatment ±1 standard error of the mean (SEM), calculated using the Mann–Whitney *U* Test. There is a significant decrease in pain score following lidocaine treatment (*n* = 16), which was not apparent post saline treatment (*n* = 8). Statistical analysis performed using the Mann–Whitney *U* test, with a *U* value of 72 (*p* = 0.0183) post lidocaine and *U* value of 30 (*p* = 0.356) post saline

Following the first urodynamic distension, mean initial pain score was reduced post void from the CC values for both the lidocaine and saline groups. This was further significantly reduced following a 20-min treatment with intravesically administered lidocaine: Z-score 2.0917, *p* 0.018. There was no significant difference in pain scores post saline treatment: Z-score 0.367, *p* 0.356 (Fig. 1). Of note, there was no difference in frequency distribution of pain score post void between groups prior to treatment. There was no change in bladder compliance in either group. Thus, the group as a whole exhibited a significant effect of lidocaine on pain scores but no effect of saline (Table 1).

**Table 1:** Pain Scores of individual participants

Post void, *n* = 16			Post void, *n* = 8		
Pain score: 0–10	Frequency	% distribution	Pain score: 0–10	Frequency	% distribution
0	3	18.8%	0	3	37.5%
1	1	6.3%	1	1	12.5%
2	3	18.8%	2	1	12.5%
3	2	12.5%	3	0	0%
4	4	25.0%	4	1	12.5%
5	1	6.3%	5	1	12.5%
6	1	6.3%	6	1	12.5%
7	0	0%	7	0	0%
8	0	0%	8	0	0%
9	0	0%	9	0	0%
10	1	6.3%	10	0	0%
Mean = 3.125			Mean = 2.25		
STD = 2.6			STD = 2.4		
Post lidocaine, *n* = 16			Post saline, *n* = 8		
Pain score: 0–10	Frequency	% distribution	Pain score: 0–10	Frequency	% distribution
0	9	56.3%	0	3	37.5%
1	0	0%	1	1	12.5%
2	3	12.5%	2	2	25%
3	2	12.5%	3	0	0%
4	1	6.3%	4	2	25%
5	0	0%	5	0	0%
6	0	0%	6	0	0%
7	0	0%	7	0	0%
8	0	0%	8	0	0%
9	1	6.3%	9	0	0%
10	0	0%	10	0	0%
Mean = 1.562			Mean = 1.625		
STD = 2.4			STD = 1.7		

Pain Score is measured on an 11-point numeric rating pain scale at MCC, CC, post void and following either lidocaine or saline treatment. There is a statistically significant decrease in pain score post lidocaine treatment, which is not seen following saline treatment. Statistical test; Mann Whitney U test. MCC = Maximal cystometric capacity, CC = Cystometric capacity

### Urodynamic volumes

**Fig. 2:**
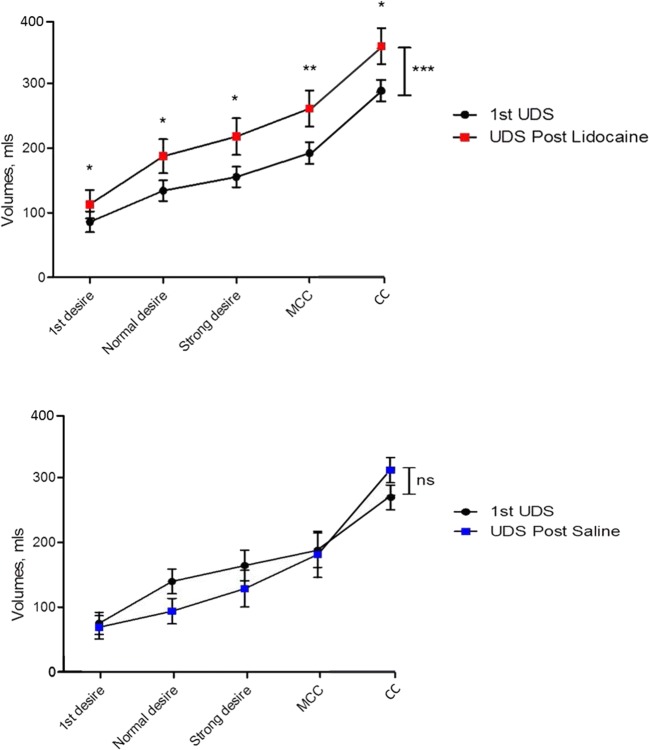
Mean volume for each sensation felt ± 1 standard error of the mean (SEM) for the first urodynamic examination and for the lidocaine and saline treatment urodynamics. Significance for each sensation volume was calculated using paired Student’s *t* test pre- and post treatment, and for the difference between urodynamic examinations using two-way analysis of variance (ANOVA). *UDS* urodynamic, *MCC* maximal cyctometric capacity, *CC* cystometric capacity

There was a statistically significant volume increase in all urodynamic sensation volumes after alkalinised lidocaine treatment: first desire, normal desire, strong desire, MCC and CC. In contrast, there was no statistically significant difference in any urodynamic parameter following saline treatment (Fig. 2).

### Individual pain score analysis

**Fig. 3:**
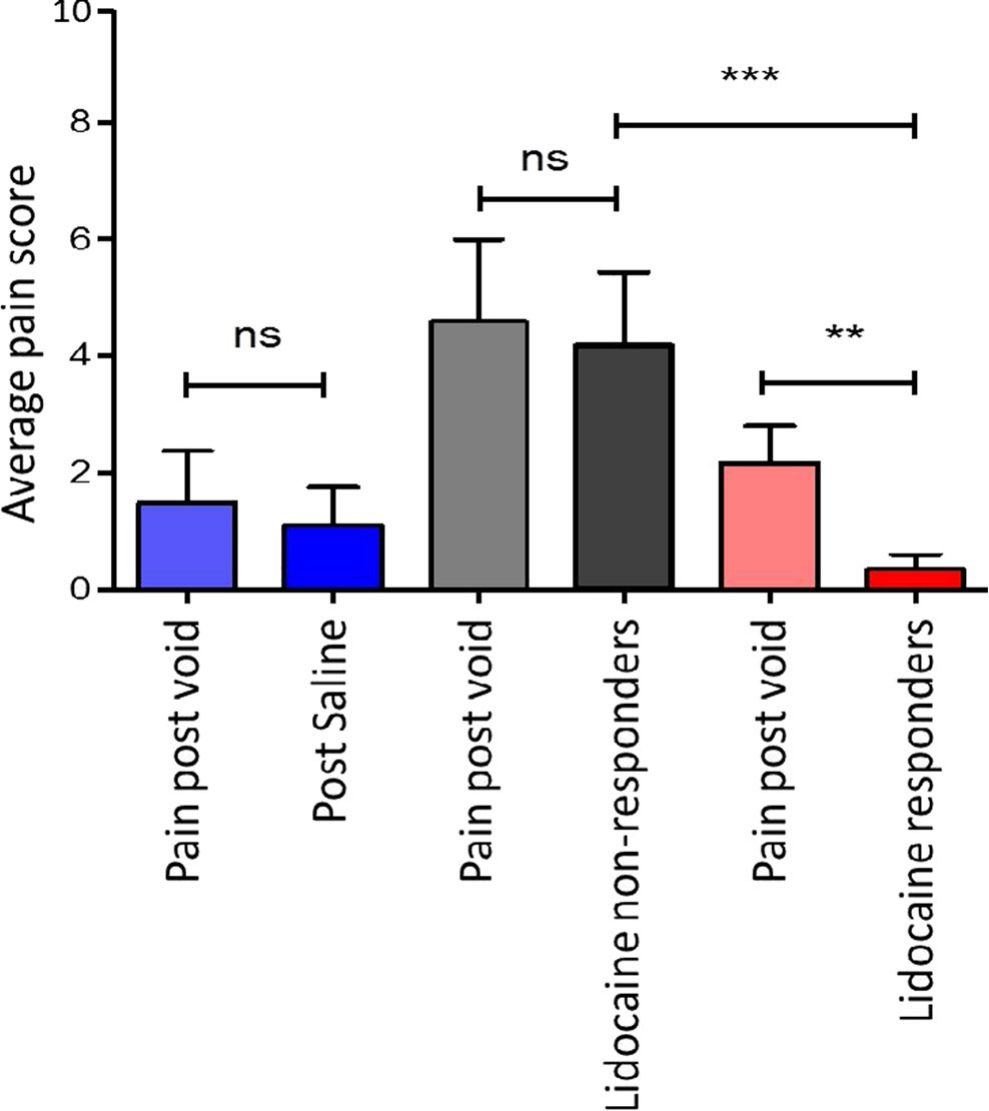
Mean numeric pain scores for each group following either lidocaine or saline treatment. The lidocaine treatment group are divided into responders and non-responders. Significance calculated using two-way analysis of variance (ANOVA) with Bonferroni multiple test comparison. ** *p* < 0.01, ***p < 0.001, *ns* non-significant.

Individual pain scores to determine participant response to alkalinised lidocaine was evaluated. Five participants had either no change or less than a 50% reduction in pain score following alkalinised lidocaine, indicating bladder instillation did not significantly reduce their reported pain. This group were designated lidocaine non-responders (Fig. 3).

### Lidocaine responders versus non-responders

**Fig. 4:**
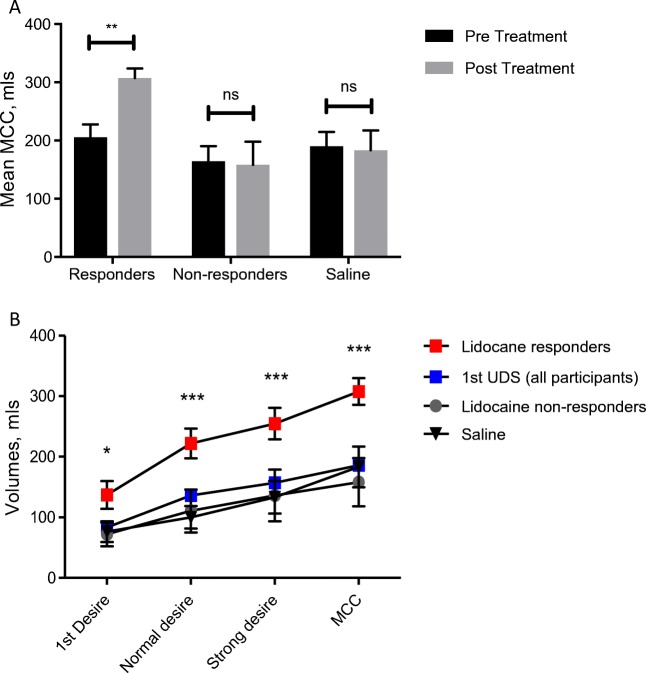
**a** Mean bladder capacity as perceived by participants in pre- and post treatment ±1 standard error of the mean (SEM). **b** First urodynamic results (blue) and subsequent distension volumes following treatment. Significance calculated using two-way analysis of variance (ANOVA) with Bonferroni multiple test comparison. *MCC* maximum cystometric capacity. *p < 0.05, ***p < 0.001

During the second urodynamic distension, mean MCC was significantly increased in those who responded to lidocaine treatment (*p* = 0.007). This was not observed in the five non-responders (*p* = 0.89) (Fig. 4a). Unlike responders, in non-responders, all urodynamic parameters and volumes were unaffected, but followed the urodynamic dissension pattern of participants post-saline instillation (Fig. 4b). On cystoscopic examination, there was no difference in disease severity in non-responders versus responders. Glomerulation, petechial haemorrhage and bladder hyperaemia were present in all participants, and there was no difference in bladder capacity in either group.

### Quality of life and central sensitivity index

**Fig. 5:**
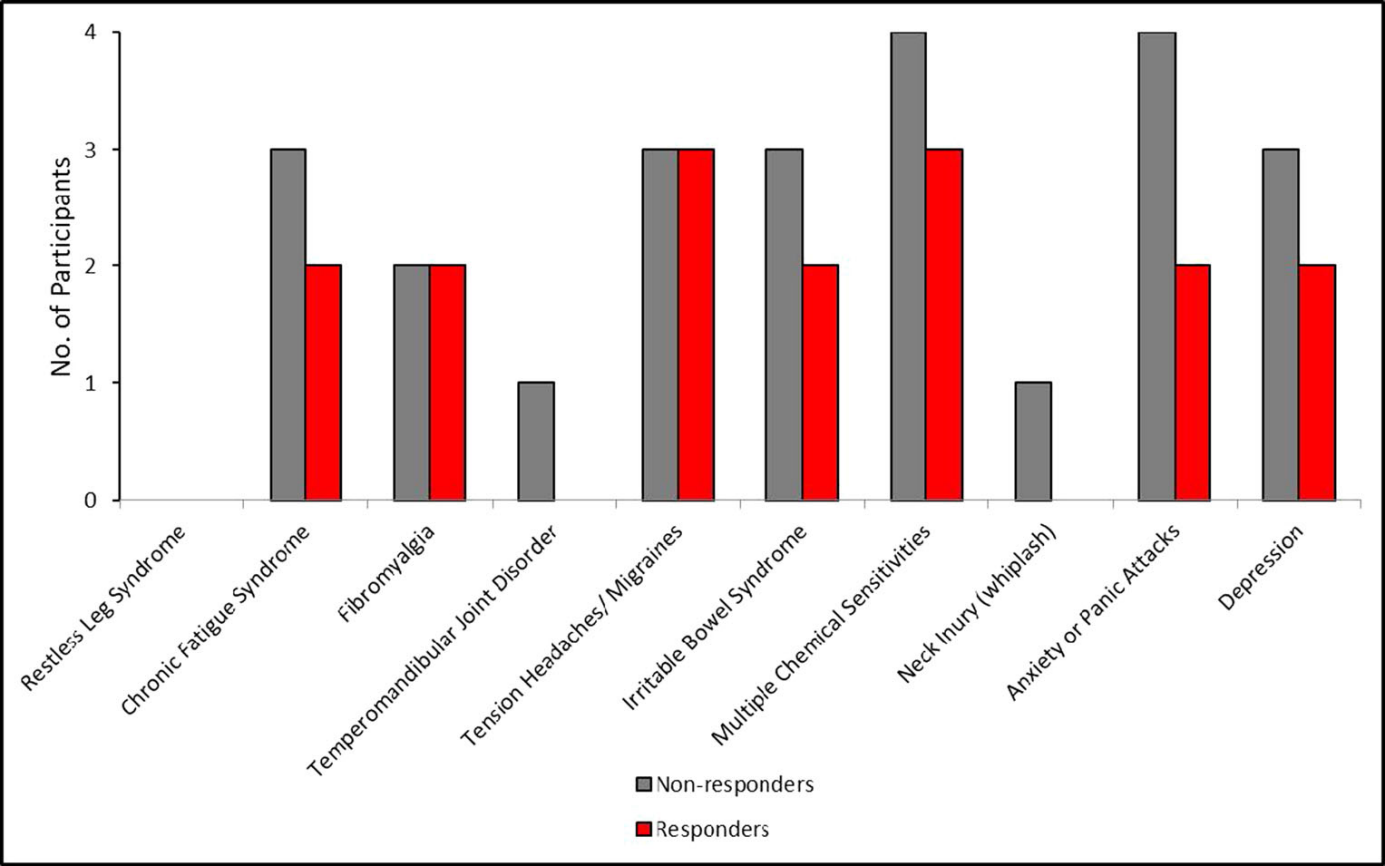
Lidocaine responders (red) versus non-responders (grey) and the presence or absence of concomitant central sensitisation syndromes (CSS) (disease). Non-responders are significantly more likely to have concomitant CSS than responders. Odds ratio (OR) = 5.423, 95% confidence interval (CI) = 2.517–11.68, *p* = < 0.0001

Non-responders to alkalinised lidocaine reported a significantly worse QoL than responders on the KHQ: mean response 1.83 and 0.6, respectively; *p* = 0.028. In addition, they had a significantly higher mean score on pain bother: 3.0 and 2.31, respectively; *p* = 0.044 (Appendix 5). Review of the CSI questionnaire revealed that non-responders were 2.7 times more likely to have other chronic pain syndromes than responders: relative risk (RR) 2.769, 95% CI 1.81–4.23, *p* < 0.0001; number needed to treat (non-responders) 2.609, 95% CI 4.377–1.858. Total concomitant CSS diagnoses were made in 24 lidocaine non-responders versus 16 in the responders (Fig. 5). Thus, non-responders were significantly more likely to have other central sensitisation-related illnesses than responders.

## Discussion

This study was aimed at evaluating the effect of intravesically administered local anaesthetics on perceived pain and urodynamic parameters in patients with BPS. Urodynamic distension and participant blinding was used to assess the importance of mechanisms underlying the pain. The study hypothesis was that patients with centrally mediated disease would not respond to local anaesthetic instillation into the bladder. A total of five participants, who did not respond to lidocaine treatment, were identified. They had more severe disease symptomatology and a higher tendency towards CSS than those who responded. These results support the hypothesis that although BPS is a disease of peripheral origin, there exists a group for whom there is a combination of both peripherally and centrally mediated pain-signalling mechanisms and a minority in whom central mechanisms dominate. For these women, it is likely that pain processing has become centralised (i.e. dominated by central pathologies in sensory processing), thus rendering peripheral treatment strategies ineffective.

Though patient reports remain valuable, it is open to subjective bias and inaccuracies due to misunderstanding the protocol or requirements. Patient reports in studies involving use of analgaesics or local anaesthetics are also open to participant performance bias and the placebo effect [14]. For that reason, use of urodynamics in this study removes participant performance or reporting bias and reduces variability in reporting. This represents the main strength of this study. Urodynamics were also used for BPS diagnosis, with patients (*n* = 4) with no urodynamic features of BPS being excluded. This was done to reduce the risk of studying patients with presumed bladder pain due to extra-urinary disease. Subsequently, these four patients had cystoscopies, which additionally confirmed no evidence of BPS.

Intravesically administrated lidocaine is one way to assess the relative contribution of peripheral versus central pathologies in BPS patients. It is a non-selective voltage-gated sodium channel blocker that blocks all nerve fibres, including motor, sensory and autonomic fibres, consequently reducing neuronal depolarisation [15, 16]. Thus, the net effect of lidocaine is complete sensory and motor block, including paralysis, loss of autonomic function and loss of all sensations. It remains the most powerful way of blocking pain while retaining consciousness. In the bladder, this nerve blockade means modulation of detrusor nerves, making lidocaine effective in the treatment of BPS. It has been used previously in the investigation and treatment of BPS. However, the effects of its use are mainly subjective, relying on patient reports of decreased pain and urinary frequency [17, 18]. Those reports are also subject to placebo effects [19, 20]. Accordingly, combining the predictable and known effects of lidocaine with objective bladder function testing using urodynamics is a unique feature of this study.

Lidocaine is a weak base provided in an acidic aqueous solution. Following tissue injection, the anaesthetic is buffered by the surrounding tissue and converted to the lipid-soluble base form, allowing for tissue penetration [21]. With intravesical instillation, this buffering by the tissue does not occur, as urine is acidic, and most of the local anaesthetic remains trapped in the urine in the acidic state. Lidocaine alkalinised with sodium bicarbonate to aid tissue penetration was used. This buffers the urine, allowing for conversion to the base form and subsequent enhanced tissue penetration [22]. Serum lidocaine levels are shown to be significantly higher with intravesically administered alkalinised lidocaine than the non-alkalinised form [23]. Alkalinised lidocaine is reported to have tissue absorption sufficient to block the sensory neuron within the submucosal plexus of the bladder [24]. Adequate lidocaine absorption can therefore be expected, excluding the uncertainty of non-response due to insufficient lidocaine serum levels.

Central sensitisation is the “augmentation of responsiveness of central pain-signalling neurons to inputs from low-threshold mechanoreceptors” [25]. Chronic pain syndromes in which central sensitisation is suggested to play a role in include fibromyalgia, vulvodynia, chronic pelvic pain, irritable bowel syndrome and migraine. These conditions are mutually associated, with the presence of one syndrome accompanying another. Together, they are termed CSS, describing a group of medically indistinct syndromes with a pathology in which central sensitisation plays a significant role [26]. Use of the central sensitisation inventory in this study defined the presence of a higher incidence of other CSS in those not responding to alkalinised lidocaine compared with those who responded to treatment. These findings are similar to the published literature evaluating the role of central sensitisation in BPS pathology [27]. Correlation of CSS scores to urodynamic findings represents another strength of this study.

Phantom pain is a sensation of pain described by the individual as related to a limb or organ that is not physically present. This, too, is hypothesised to be as a result of central sensitisation mechanisms [28]. It occurs in 80–100% of amputees and is also present postsurgical removal of viscera. Patients with BPS occasionally complain of phantom bladder pain or sensations of bladder fullness post cystectomy or urinary diversion [4, 5]. Thus, attempts at eliminating BPS pain using either local anaesthetics or surgical excision of the bladder will not relieve pain perception if there is pain centralisation. With central sensitisation, although it feels as if the pain originates in the periphery, it is actually a manifestation of abnormal sensory processing within the CNS. It is possible that the lack of response to alkalinised lidocaine treatment in combination with the increased prevalence of other CSS in this group is due to pain-related factors remote from the periphery, such as central sensitisation. Speculation is drawn about the plausible role of central sensitisation in BPS.

ESSIC criteria for the diagnosis of BPS recommends performing cystoscopy with hydrodistension to confirm BPS and rule out any confusable diseases of the lower urinary tract [29]. All recruited participants were referred for cystoscopy where BPS/IC was diagnosed (as evidenced by petechial haemorrhages). Bladder biopsy is not a prerequisite for diagnosis of BPS and was not performed. However, other pelvic pain disorders, such as endometriosis or pelvic inflammatory disease, could account for the symptoms described and present as pain. These cannot be excluded without diagnostic laparoscopy. Thus, meticulous history taking to exclude patients whose presenting symptoms were suggestive of extra-urinary disease was made a priority of recruitment and inclusion in the study. Of course, with the presence of inadvertent concomitant extra-urinary pain disorders, intravesically administered lidocaine is not expected to alleviate the pain.

We additionally used urodynamics for identification of BPS patients. Bladder compliance can either be normal or low. Low compliance, due to changes in viscoelastic properties of the bladder secondary to chronic inflammation, can be attributed to certain clinically relevant conditions, such as neurogenic bladder, radiation cystitis and BPS. All women with a history of neurological disorders that may predispose to neurogenic bladder, as well as women with radiation cystitis, were excluded at recruitment. Consequently, the finding of low compliance in this cohort, backed up by pain history and reduced bladder volumes, is further suggestive of BPS. Of note, only one participant had an MCC > 300 ml. She, however, had a pain score of 10 at MCC, low compliance and reduced sensations. She also had cystoscopic evidence of BPS and was thus included in the analysis.

To identify lidocaine responders versus non-responders, the difference in numeric pain scores pre- and post-lidocaine treatment was computed. Previous studies used a 50% reduction in pain score as a cutoff measure of non-responders [19, 23]. This method was therefore used in this study, as it appeared reliable and reproducible and removed the bias of choosing an arbitrary pain score. In addition, each individual’s experience of pain is different and unique to that person’s experiences; thus, a fixed-reference pain score does not represent a meaningful pain threshold for all individuals. Average pain score at cystometric capacity was recorded as 9.8 and reduced post void to 3.1. This is due to the relief gained by bladder emptying on the BPS pathology. However, one limitation of this study was that ten patients who had severe BPS, with a pain score >5 on the 10-point scale post-void, were unwilling to have second urodynamic distension. The study was hence limited to examining patients with moderate instead of severe disease. However, as the literature suggests, phantom pain due to pain centralisation is not only present with severe disease; it can also occur with pain chronicity and following psychological trauma. It was consequently deemed appropriate to continue analysis in this moderate-pain group, as the phenomenon of pain centralisation nonetheless remained valid. Exclusion of these patients also meant there was a smaller number of participants for final analysis. However, statistical analysis reached significance despite these small numbers. Previous publications using lidocaine in BPS have employed a similar number of participants, with sufficient power to demonstrate statistical significance [19, 30].

The results of this study allow for consideration of the pathophysiology of pain associated with BPS. Characterising patients with BPS in this manner can aid in streamlining treatment strategies. Patients with central dominant pain may receive central therapies earlier using a multidisciplinary approach to pain management, while those with peripheral dominant disease may be managed with peripheral bladder therapies. Additionally, when all other treatments have failed, prior to radical management options such as cystectomy or urinary diversion for intractable pain, this simple technique may distinguish those for whom phantom bladder pain may persist postoperatively.

## Conclusion

Lidocaine use, in this small observational study, allowed for the evaluation of BPS from a pain perspective. The findings of this study have important implications for day-to-day practice, because by using this simple technique to stratify patients into central or peripheral dominant pain mechanisms or a combination of both, treatment strategies can be more focused, bypassing the routine stepwise trial-and-error approach commonly used in BPS management. A study evaluating the central neural response to invoked bladder pain in BPS patients, such as those using functional magnetic resonance imaging, is warranted to confirm the role of central mechanisms in the BPS pathology. The promising early findings from this study introduces a setting in which additional recruitment and further research will provide confirmation.

## Electronic supplementary material Please confirm if appendix figures was captured correctly.I do not have access to the appendix figures.

ESM 1 (PDF 17 kb)

ESM 2 (PDF 269 kb)

ESM 3 (PDF 102 kb)

ESM 4 (DOCX 39 kb)

ESM 5 (DOCX 68 kb)
