# Supplementary material for: Prospective comparative study of the effects of lidocaine on urodynamic and sensory parameters in bladder pain syndrome
Source: Int Urogynecol J. 2019 Mar 14;30(8):1293–301. doi: 10.1007/s00192-019-03892-2 (PMC6647211; doi:10.1007/s00192-019-03892-2)
Supplement: Supplementary file 1 — (PDF 17 kb) [file 192_2019_3892_MOESM1_ESM.pdf]

THE KING'S HEALTH QUESTIONNAIRE

Your name:

Your Kaiser medical record number:

1. How would you describe your health at the present?

Please tick one answer

- **Very good** ⬭
- **Good** ⬭
- **Fair** ⬭
- **Poor** ⬭
- **Very poor** ⬭

2. How much do you think your bladder problem affects your life?

Please tick one answer

- **Not at all** ⬭
- **A little** ⬭
- **Moderately** ⬭
- **A lot** ⬭

Please turn the page

Below are some daily activities that can be affected by bladder problems.
How much does your bladder problem affect you?

We would like you to answer every question. Simply tick the box that applies to you

**3. ROLE LIMITATIONS**	**1** Not at all	**2** Slightly	**3** Moderately	**4** A lot
**A.** Does your bladder problem affect your household tasks? (cleaning, shopping etc)	⬭	⬭	⬭	⬭
**B.** Does your bladder problem affect your job, or your normal daily activities outside the home?	⬭	⬭	⬭	⬭

**4. PHYSICAL/SOCIAL LIMITATION**	**1** Not at all	**2** Slightly	**3** Moderately	**4** A lot
**A** Does your bladder problem affect your physical activities (e.g. going for a walk, running, sport, gym etc)?	⬭	⬭	⬭	⬭
**B.** Does your bladder problem affect your ability to travel?	⬭	⬭	⬭	⬭
**C.** Does your bladder problem limit your social life?	⬭	⬭	⬭	⬭
**D.** Does your bladder problem limit your ability to see and visit friends?	⬭	⬭	⬭	⬭

**5. PERSONAL RELATIONSHIPS**	**0** Not Applicable	**1** Not at all	**2** Slightly	**3** Moderately	**4** A lot
**A.** Does your bladder problem affect your relationship with your partner?	⬭	⬭	⬭	⬭	⬭
**B.** Does your bladder problem affect your sex life?	⬭	⬭	⬭	⬭	⬭
**C.** Does your bladder problem affect your family life?	⬭	⬭	⬭	⬭	⬭

**6. EMOTIONS**	1<br>Not at all	2<br>Slightly	3<br>Moderately	4<br>Very much
**A.** Does your bladder problem make you feel depressed?	⬭	⬭	⬭	⬭
**B.** Does your bladder problem make you feel anxious or nervous?	⬭	⬭	⬭	⬭
**C.** Does your bladder problem make you feel bad about yourself?	⬭	⬭	⬭	⬭

**7.SLEEP/ENERGY**	1<br>Never	2<br>Sometimes	3<br>Often	4<br>All the time
**A.** Does your bladder problem affect your sleep?	⬭	⬭	⬭	⬭
**B.** Does your bladder problem make you feel worn out and tired ?	⬭	⬭	⬭	⬭

8.Do you do any of the following? **If so how much?**

	1<br>Never	2<br>Sometimes	3<br>Often	4<br>All the time
**A.** Wear pads to keep dry?	⬭	⬭	⬭	⬭
**B.** Be careful how much fluid you drink ?	⬭	⬭	⬭	⬭
**C.** Change your underclothes because they get wet?	⬭	⬭	⬭	⬭
**D.** Worry in case you smell?	⬭	⬭	⬭	⬭

We would like to know what your bladder problems are and how much they affect you ? From the list below choose only those problems that you have at present. Leave out those that don't apply to you.

How much do they affect you?

FREQUENCY: going to the toilet very often

1. A little ○ | **2.Moderately** ○ | **3. A lot** ○

NOCTURIA: getting up at night to pass urine

1. A little ○ | **2.Moderately** ○ | **3. A lot** ○

URGENCY: a strong and difficult to control desire to pass urine

1. A little ○ | **2.Moderately** ○ | **3. A lot** ○

URGE INCONTINENCE: urinary leakage associated with a strong desire to pass urine

1. A little ○ | **2.Moderately** ○ | **3. A lot** ○

STRESS INCONTINENCE: urinary leakage with physical activity eg. coughing, running

1. A little ○ | **2.Moderately** ○ | **3. A lot** ○

NOCTURNAL ENURESIS: wetting the bed at night

1. A little ○ | **2.Moderately** ○ | **3. A lot** ○

INTERCOURSE INCONTINENCE: urinary leakage with sexual intercourse

1. A little ○ | **2.Moderately** ○ | **3. A lot** ○

WATERWORKS INFECTIONS

1. A little ○ | **2.Moderately** ○ | **3. A lot** ○

BLADDER PAIN

1. A little ○ | **2.Moderately** ○ | **3. A lot** ○

Thank You For Your Time
