# Supplementary material for: Prospective comparative study of the effects of lidocaine on urodynamic and sensory parameters in bladder pain syndrome
Source: Int Urogynecol J. 2019 Mar 14;30(8):1293–301. doi: 10.1007/s00192-019-03892-2 (PMC6647211; doi:10.1007/s00192-019-03892-2)
Supplement: Supplementary file 2 — (PDF 269 kb) [file 192_2019_3892_MOESM2_ESM.pdf]

Appendix 2: *Adapted from Mayer et al, 2012*. The Central Sensitization Inventory: Part B.

Have you been diagnosed by a doctor with any of the following disorders? Please check the box to the right for each diagnosis and write the year of the diagnosis				
		NO	YES	Year Diagnosed
1	Restless Leg Syndrome			
2	Chronic Fatigue Syndrome			
3	Fibromyalgia			
4	Temporomandibular Joint Disorder (TMJ)			
5	Tension Headaches / Migraines			
6	Irritable Bowel Syndrome			
7	Multiple Chemical Sensitivities			
8	Neck Injury (including whiplash)			
9	Anxiety or Panic attacks			
10	Depression			
