# Supplementary figures and images for: Prospective comparative study of the effects of lidocaine on urodynamic and sensory parameters in bladder pain syndrome
Source: Int Urogynecol J. 2019 Mar 14;30(8):1293–301. doi: 10.1007/s00192-019-03892-2 (PMC6647211; doi:10.1007/s00192-019-03892-2)

### Appendix 3: Numeric pain rating scale

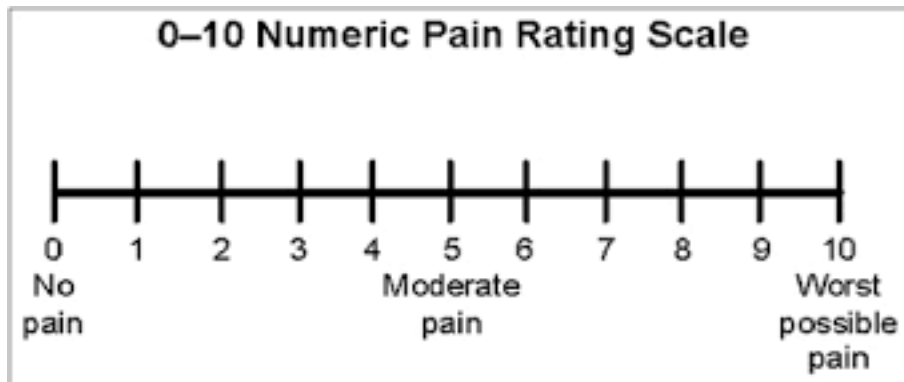

Supplement: Supplementary file 3 — (PDF 102 kb) [file 192_2019_3892_MOESM3_ESM.pdf]
