# Supplementary material for: Prospective comparative study of the effects of lidocaine on urodynamic and sensory parameters in bladder pain syndrome
Source: Int Urogynecol J. 2019 Mar 14;30(8):1293–301. doi: 10.1007/s00192-019-03892-2 (PMC6647211; doi:10.1007/s00192-019-03892-2)
Supplement: Supplementary file 4 — (DOCX 39 kb) [file 192_2019_3892_MOESM4_ESM.docx]

Appendix: Frequency distribution of pain scores for all participants at Cystometric capacity, post void and post lidocaine or saline intravesical instillation. CC = Cystometric capacity
