# Supplementary material for: Prospective comparative study of the effects of lidocaine on urodynamic and sensory parameters in bladder pain syndrome
Source: Int Urogynecol J. 2019 Mar 14;30(8):1293–301. doi: 10.1007/s00192-019-03892-2 (PMC6647211; doi:10.1007/s00192-019-03892-2)
Supplement: Supplementary file 5 — (DOCX 68 kb) [file 192_2019_3892_MOESM5_ESM.docx]

Appendix 5: Correlation of pain score with KHQ symptoms total score. There is a positive relationship between the severity of pain reported by the lidocaine non-responders and a poorer quality of life as assessed with the KHQ. This is not seen with the lidocaine responders: correlation coefficient 0.665 versus -0.489 respectively.
